# Stable conditional expression and effect of C/EBPβ-LIP in adipocytes using the pSLIK system

**DOI:** 10.1530/JME-13-0029

**Published:** 2013-08

**Authors:** Cristina L Esteves, Val Kelly, Valérie Bégay, Simon G Lillico, Achim Leutz, Jonathan R Seckl, Karen E Chapman

**Affiliations:** 1 Endocrinology UnitQueen's Medical Research Institute, University/BHF Centre for Cardiovascular Science, The University of Edinburgh47 Little France Crescent, Edinburgh, EH16 4TJUK; 2 Max Delbrüeck Centre for Molecular MedicineRobert-Rössle-Straße 1013125, BerlinGermany; 3 Division of Developmental BiologyThe Roslin Institute, The University of EdinburghEaster Bush Campus, Midlothian, EH25 9RGUK

**Keywords:** adipocyte, stable transfection, pSLIK, C/EBPβ-LIP

## Abstract

Murine 3T3-L1 adipocytes are widely used as a cellular model of obesity. However, whereas transfection of 3T3-L1 preadipocytes is straightforward, ectopic gene expression in mature 3T3-L1 adipocytes has proved challenging. Here, we used the pSLIK vector system to generate stable doxycycline-inducible expression of the liver-enriched inhibitor protein isoform of CCAAT/enhancer binding protein β (CEPB (C/EBPβ-LIP)) in fully differentiated 3T3-L1 adipocytes. Because overexpression of C/EBPβ-LIP impairs adipocyte differentiation, the C/EBPβ-LIP construct was first integrated in 3T3-L1 preadipocytes but expression was induced only when adipocytes were fully differentiated. Increased C/EBPβ-LIP in mature adipocytes down-regulated C/EBPβ target genes including 11β-hydroxysteroid dehydrogenase type 1, phosphoenolpyruvate carboxykinase and fatty acid binding protein 4 but had no effect on asparagine synthetase, demonstrating that transcriptional down-regulation by C/EBPβ-LIP in 3T3-L1 adipocytes is not a general effect. Importantly, these genes were modulated in a similar manner in adipose tissue of mice with genetically increased C/EBPβ-LIP levels. The use of the pSLIK system to conditionally express transgenes in 3T3-L1 cells could be a valuable tool to dissect adipocyte physiology.

## Introduction

First developed almost 40 years ago from Swiss mouse embryo cells, 3T3-L1 adipocytes (Otto & Lane 2005, Rosen & MacDougald 2006) have proved to be an invaluable tool, advancing our understanding of cellular mechanisms underlying obesity-related disorders. However, introduction of nucleic acids and, consequently, the manipulation of genes in fully differentiated 3T3-L1 adipocytes are difficult to achieve. pSLIK plasmids, encoding a tetracycline-regulated transcriptional unit in a lentivirus-based vector backbone, were originally designed to facilitate stable transduction of microRNA-like short hairpin RNAs into RAW264.7 cells (Shin *et al*. 2006) but have not been tested in adipocytes.

The transcription factor CCAAT/enhancer binding protein β (CEBPB (C/EBPβ)) is produced as three major isoforms resulting from differential use of in-frame translation start sites or from proteolysis (Ossipow *et al*. 1993, Welm *et al*. 1999, Calkhoven *et al*. 2000); the 38 and 35 kDa liver-enriched activator protein isoforms (C/EBPβ-LAP* and C/EBPβ-LAP respectively) and the 20 kDa liver-enriched inhibitor protein (LIP; C/EBPβ-LIP). C/EBPβ-LIP contains the carboxyl-terminal dimerisation and DNA binding domains but lacks the amino-terminal activation domain of C/EBPβ-LAP (Ramji & Foka 2002). Consequently, C/EBPβ-LIP represses transcription by forming either homodimers or heterodimers with the C/EBPβ-LAP isoforms or other C/EBPs (Descombes *et al*. 1990, Calkhoven *et al*. 2000) and manipulation of C/EBPβ-LIP levels may change C/EBPβ-LIP:LAP ratio. However, the majority of reports do not differentiate between C/EBPβ isoforms, and genetic disruption of the encoding gene eliminates production of all isoforms. While C/EBPβ is well recognised as a major player in adipocyte differentiation and metabolism, the role of the specific isoforms, and especially the C/EBPβ-LIP isoform, is less clear. We have recently shown that genetic manipulation of levels of C/EBPβ-LIP *in vivo* changes adipose tissue expression of the C/EBPβ target gene that encodes the glucocorticoid amplifying enzyme 11β-hydroxysteroid dehydrogenase type 1 (*Hsd11b1* (11β-HSD1); Seckl *et al*. 2004, Esteves *et al*. 2012). However, exploration of mechanisms in 3T3-L1 adipocytes is hindered by the impairment of preadipocyte differentiation by C/EBPβ-LIP (Yeh *et al*. 1995, Payne *et al*. 2007, Abdou *et al*. 2011).

Here, we have used the pSLIK system to generate stable inducible expression of C/EBPβ-LIP in 3T3-L1 adipocytes. Increased levels of C/EBPβ-LIP in 3T3-L1 adipocytes decreased the expression of the metabolically relevant C/EBPβ target genes encoding phosphoenolpyruvate carboxykinase (*Pck1* (PEPCK)), fatty acid binding protein 4 (*Fabp4* (FABP4)) and asparagine synthetase in a manner similar to manipulation of C/EBPβ-LIP *in vivo*.

## Materials and methods

### Ethics statement

All animal experiments were conducted in strict accordance with internationally accepted standards of humane animal care following prior approval by the Institutional Animal Care and Use Committee in Berlin, Germany.

### Cells and animals

Murine 3T3-L1 preadipocytes (American Type Culture Collection (ATCC)) were maintained in DMEM (Lonza, Cambridge, UK) supplemented with newborn calf serum (10%; Lonza), unless otherwise stated. Adult male C/EBPβ mutant mice, heterozygous C/EBPβ^(^^+^^/L)^, homozygous C/EBPβ^Δ^^uORF^ and respective wild-type (WT) littermate control mice (*n*=6–7/group) were generated as described previously (Smink *et al*. 2009, Wethmar *et al*. 2010).

### Stable transfection of 3T3-L1 preadipocytes

C/EBPβ-LIP DNA sequence was excised from pMSV-C/EBPβ (a gift from S L McKnight and W-C Yeh), ligated to an internal ribosome entry site (IRES) to allow for downstream GFP expression and the resulting C/EBPβ-LIP-IRES construct was cloned into pMBA-266. C/EBPβ-LIP and control (empty) pSLIK vectors (Shin *et al*. 2006) were generated by gateway recombination between a pEN (MBA-266; ATCC) entry vector harbouring C/EBPβ-LIP or empty vector and the pSLIK-Neo destination vector (MBA-235; ATCC). 3T3-L1 preadipocytes were transfected with control or C/EBPβ-LIP-expressing pSLIK vectors using Lipofectamine 2000 (Invitrogen), followed by ≥15-day selection of cells resistant to 1 mg/ml geneticin (Invitrogen). Transfection efficiency was high (data not shown) and a total pool of transfected preadipocytes was obtained. Both control and C/EBPβ-LIP transfectants expressed GFP in the presence of doxycycline (DOX; 1 μg/ml; Sigma) and were further selected by cell sorting in the presence of DOX (FACSVantage, BD Biosciences, San Jose, CA, USA). Preadipocytes were maintained throughout in geneticin.

### Differentiation of 3T3-L1 preadipocytes into adipocytes

3T3-L1 preadipocytes, cultured in the absence of DOX, harbouring C/EBPβ-LIP or control empty vector, were induced to differentiate into mature adipocytes as described previously (Napolitano *et al*. 1998). Cells were grown to confluence, and 2 days later, they were induced to differentiate in DMEM supplemented with 10% FCS, 0.5 μM dexamethasone, 500 μM 3-isobutyl-1-methylxanthine and 5 μg/ml insulin for 2 days. Thereafter, 3T3-L1 cell differentiation continued in medium supplemented with 5 μg/ml insulin alone. Experiments were performed 8–12 days following induction of differentiation, unless otherwise stated. DOX (1 μg/ml) was added to the medium in the experiments designed to induce C/EBPβ-LIP.

### Western blot analysis

Cells were harvested in lysis buffer (0.125 M Tris–HCL, pH 6.8, 2% SDS and 10% glycerol) in the presence of a protease inhibitor cocktail (P2714; Sigma) and heated at 100 °C. Electrophoresis was carried out on 4–12% NuPage Bis–Tris gels (Invitrogen). After transfer, blots were probed with antibodies to C/EBPβ (1:500 dilution of stock 200 μg/ml; Santa Cruz Biotechnology, Inc.) and β-tubulin (1:10 000 dilution; Chemicon/Millipore, Watford, UK). Secondary antibodies were anti-rabbit IgG-HRP (1:2000 dilution from stock 400 μg/ml; Santa Cruz Biotechnology, Inc.), anti-mouse IgG-HRP (1:4000 dilution; Cell Signaling, Danvers, MA, USA), anti-rabbit IgG Alexa Fluor 700 (Invitrogen) or anti-mouse IgG 488 Conjugated (Rockland Immunochemicals, Inc., Gilbertsville, PA, USA). The resulting bands were visualised on X-ray films developed in a Konica SRX-101 X-Ray developer or using the Odyssey Infrared Imaging System, as appropriate.

### Oil Red O staining

3T3-L1 adipocytes were stained by Oil Red O as described previously (Lillie & Ashburn 1943) with modifications. Cells were immersed in isopropanol (60%), followed by the staining solution (0.04% Oil Red O in 60% isopropanol) and ammonia (1%) and then visualised using a Zeiss Axioskop microscope. Quantification of Oil Red O staining density was performed with ImageJ (NIH, Bethesda, MA, USA).

### RNA extraction and real-time PCR

Cells were harvested in TRIzol (Invitrogen) and RNA was extracted according to the manufacturer's protocol. Mouse subcutaneous adipose tissue was dissected and frozen (−70 °C) prior to RNA extraction using Qiagen RNeasy Lipid Tissue Mini kit (Qiagen). RNA (1 μg) was reverse transcribed using SuperScript III (Invitrogen), and specific mRNAs were quantified by real-time PCR using a LightCycler 480 (Roche) as described (Sai *et al*. 2008). Primer–probe sets were purchased from Applied Biosystems: C/EBPβ (Mm00843434_s1), 11β-HSD1 (Mm00476182_m1), PEPCK (Mm01247058_m1), FABP4 (Mm00445878_m1) and asparagine synthetase (Mm00803785_m1). TATA binding protein (TBP; Mm00446973_m1) was used as internal control and did not change with treatment or between groups.

### Statistical analysis

All data were analysed by Student's *t*-test or one-way ANOVA followed by *post hoc* least square difference test using SigmaStat 2.03 Statistical Software. Significance was set at *P*≤0.05.

## Results

### Conditional expression of C/EBPβ-LIP in stably transfected 3T3-L1 preadipocytes and differentiated adipocytes

**Figure 1:**
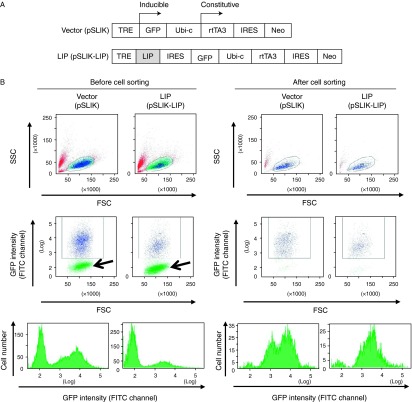
The pSLIK-C/EBPβ-LIP construct and generation of stably transfected 3T3-L1 preadipocytes. (A) Schematic representation of the pSLIK Tet-On empty vector (top; vector, pSLIK) and construct used to generate stable 3T3-L1 preadipocytes conditionally overexpressing C/EBPβ-LIP (bottom; LIP, pSLIK-C/EBPβ-LIP). C/EBPβ-LIP and GFP are co-expressed from the same transcript. The constitutively active ubiquitin c (Ubi-c) promoter drives expression of the Tet activator (rtTA3) and neomycin resistance gene (Neo; for selection). Addition of DOX causes binding of rtTA3 to the TRE/CMV promoter (TRE), inducing expression of GFP or C/EBPβ-LIP-IRES-GFP. (B) FACS analysis and sorting of 3T3-L1 preadipocytes (5×10^6^ cells) stably transfected with pSLIK-C/EBPβ-LIP (LIP) or pSLIK vector (vector) following DOX treatment (1 μg/ml, 24 h) to induce GFP. Upper panels, forward scatter (FSC) vs side scatter (SSC) dot plots, showing the populations of single cells (circled) that were analysed and sorted for GFP expression (FSC vs GFP intensity plots; middle row of panels) and histogram showing the number of cells for GFP intensity (lower panels). Middle and lower panels show the enrichment of GFP-positive cells for both pSLIK-C/EBPβ-LIP and pSLIK cells after sorting (right panels) resulting from the removal of low or non-GFP-expressing preadipocytes (arrow; left panels).

**Figure 2:**
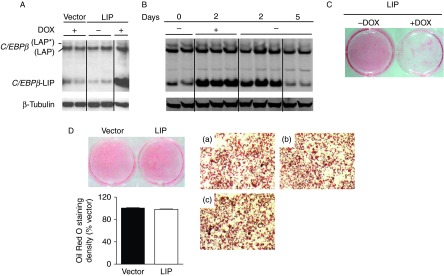
Doxycycline-inducible expression of C/EBPβ-LIP in fully differentiated adipocytes and adipocyte differentiation visualised by Oil Red O staining. (A) Western blot showing induction of C/EBPβ-LIP expression in 3T3-L1 pSLIK-C/EBPβ-LIP (LIP, +DOX) or pSLIK control vector (vector, +DOX) adipocytes. Preadipocytes were differentiated into adipocytes in DOX-free medium. Mature adipocytes were then treated with DOX for 2 days. In the absence of DOX, C/EBPβ-LIP levels were not elevated above basal endogenous levels in pSLIK-C/EBPβ-LIP adipocytes (LIP, −DOX). The blot (40 μg protein/lane) was probed with C/EBPβ antibody (38 kDa LAP*, 35 kDa LAP and 20 kDa LIP are indicated), then stripped and reprobed with β-tubulin antibody, as loading control. All samples in each membrane were analysed in the same gel, but not in adjacent lanes. (B) Western blot showing robust induction of C/EBPβ-LIP in pSLIK-C/EBPβ-LIP adipocytes, previously differentiated in DOX-free medium and then treated with DOX between days 0 and 2. Samples (50 μg protein/lane) were taken before treatment (0), 2 days after DOX-induction of C/EBPβ-LIP (2; +DOX) and 2 and 5 days following DOX withdrawal from the medium (2 and 5; −DOX). (C and D) Oil Red O staining showing lipid accumulation in differentiated adipocytes. 3T3-L1 pSLIK-C/EBPβ-LIP preadipocytes were differentiated into adipocytes in the presence (+DOX) or absence (−DOX) of DOX (C) or were differentiated in the absence of DOX and then DOX-treated for 3 days prior to Oil Red O staining (D). Panels show differentiated adipocytes; pSLIK-C/EBPβ-LIP (LIP; b), pSLIK control vector (vector; a) and untransfected 3T3-L1 adipocytes (c). Objective magnification, ×10.

To generate a vector to conditionally express C/EBPβ-LIP, the encoding cDNA was cloned and recombined in the MBA-266 and the pSLIK-Neo MBA-235 vectors, respectively, producing the pSLIK-C/EBPβ-LIP vector in which a tetracycline response element drives expression of C/EBPβ-LIP from a minimal CMV promoter in the presence of DOX (a Tet-ON system) and a contiguous IRES allows for GFP expression. Constitutive expression of the reverse Tet transactivator (rtTA3) and the neomycin resistance gene (Neo) is driven by the ubiquitin-c (Ubi-c) promoter (Fig. 1A). In order to express C/EBPβ-LIP in mature adipocytes, we first generated stably transfected 3T3-L1 preadipocytes that, in contrast to fully differentiated adipocytes, could be readily transfected with pSLIK-C/EBPβ-LIP or the ‘empty’ vector. This was highly efficient allowing selection of transfectants with geneticin as a total pool of cells, avoiding clonal differences due to plasmid integration site. To ensure that only transfected cells were used in the subsequent experiments, preadipocytes were sorted by flow cytometry for GFP expression 24 h after incubation with DOX, with yields of 26 and 56% for pSLIK-C/EBPβ-LIP and pSLIK-transfectants respectively (Fig. 1B). In the absence of DOX, only 0.9% of pSLIK-C/EBPβ-LIP and 4.3% of pSLIK preadipocytes were GFP positive, demonstrating low spontaneous expression in the transfected preadipocytes. pSLIK-C/EBPβ-LIP preadipocytes were then differentiated into mature adipocytes in DOX-free medium (to avoid the inhibitory effects of C/EBPβ-LIP upon adipocyte differentiation) and subsequent DOX treatment robustly increased cellular C/EBPβ-LIP levels in the mature adipocytes (Fig. 2A and B). Following removal of DOX from the cell medium, C/EBPβ-LIP decreased as expected, showing that it can be transiently induced (Fig. 2B). Importantly, in the absence of DOX, pSLIK-C/EBPβ-LIP adipocytes showed comparable low endogenous levels of C/EBPβ-LIP to control adipocytes harbouring the pSLIK empty vector (Fig. 2A).

### 3T3-L1 preadipocytes stably transfected with pSLIK-C/EBPβ-LIP differentiate normally into adipocytes

Consistent with previous data (Yeh *et al*. 1995, Payne *et al*. 2007, Abdou *et al*. 2011), adipocyte differentiation (demonstrated by Oil Red O staining of lipid) was markedly attenuated if C/EBPβ-LIP expression was induced in 3T3-L1 pSLIK-C/EBPβ-LIP preadipocytes prior to and during differentiation by DOX (Fig. 2C). In contrast, when pSLIK-C/EBPβ-LIP preadipocytes were differentiated in the absence of DOX, both pSLIK control and pSLIK-C/EBPβ-LIP preadipocytes differentiated normally into mature adipocytes. Induction of C/EBPβ-LIP in fully differentiated adipocytes had no effect on their differentiation status as shown by quantification of Oil Red O staining (Fig. 2D).

### Increased C/EBPβ-LIP reduces expression of 11β-HSD1, PEPCK and FABP4 but not asparagine synthetase in 3T3-L1 mature adipocytes

**Figure 3:**
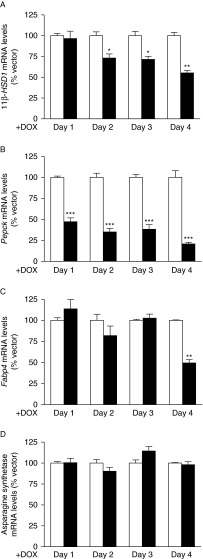
Elevated adipocyte C/EBPβ-LIP represses expression of C/EBPβ target genes but not of asparagine synthetase. Real-time PCR measurement of levels of mRNAs encoding (A) 11β-HSD1, (B) PEPCK, (C) FABP4 and (D) asparagine synthetase in 3T3-L1 pSLIK-C/EBPβ-LIP adipocytes (black bars) compared with pSLIK control adipocytes (white bars), following 1–4 days of induction with DOX. Preadipocytes were differentiated into adipocytes in DOX-free medium and DOX was added to mature adipocytes to induce C/EBPβ-LIP expression. Levels of specific mRNAs, normalised to TBP are expressed relative to levels in pSLIK adipocytes (arbitrarily set to 100%). Data are mean±s.e.m. of two independent adipocyte differentiations, each carried out in triplicate. *Significantly different from pSLIK control adipocytes. *P≤0.05, **P<0.001 and ***P<0.0001.

The transcriptional impact of increased C/EBPβ-LIP in adipocytes was examined following induction with DOX only once 3T3-L1 adipocytes were fully differentiated. Concurrent with the increase in C/EBPβ-LIP expression, and consequently C/EBPβ-LIP:LAP ratio, DOX reduced 11β-HSD1 and PEPCK mRNA levels in 3T3-L1 pSLIK-C/EBPβ-LIP adipocytes compared with control cells (Fig. 3A and B). FABP4 mRNA levels were decreased following 4 days of DOX treatment but asparagine synthetase mRNA levels were unaffected (Fig. 3C and D).

### Modulation of C/EBPβ-LIP levels in adipose tissue *in vivo* changes the expression of PEPCK and FABP4

**Figure 4:**
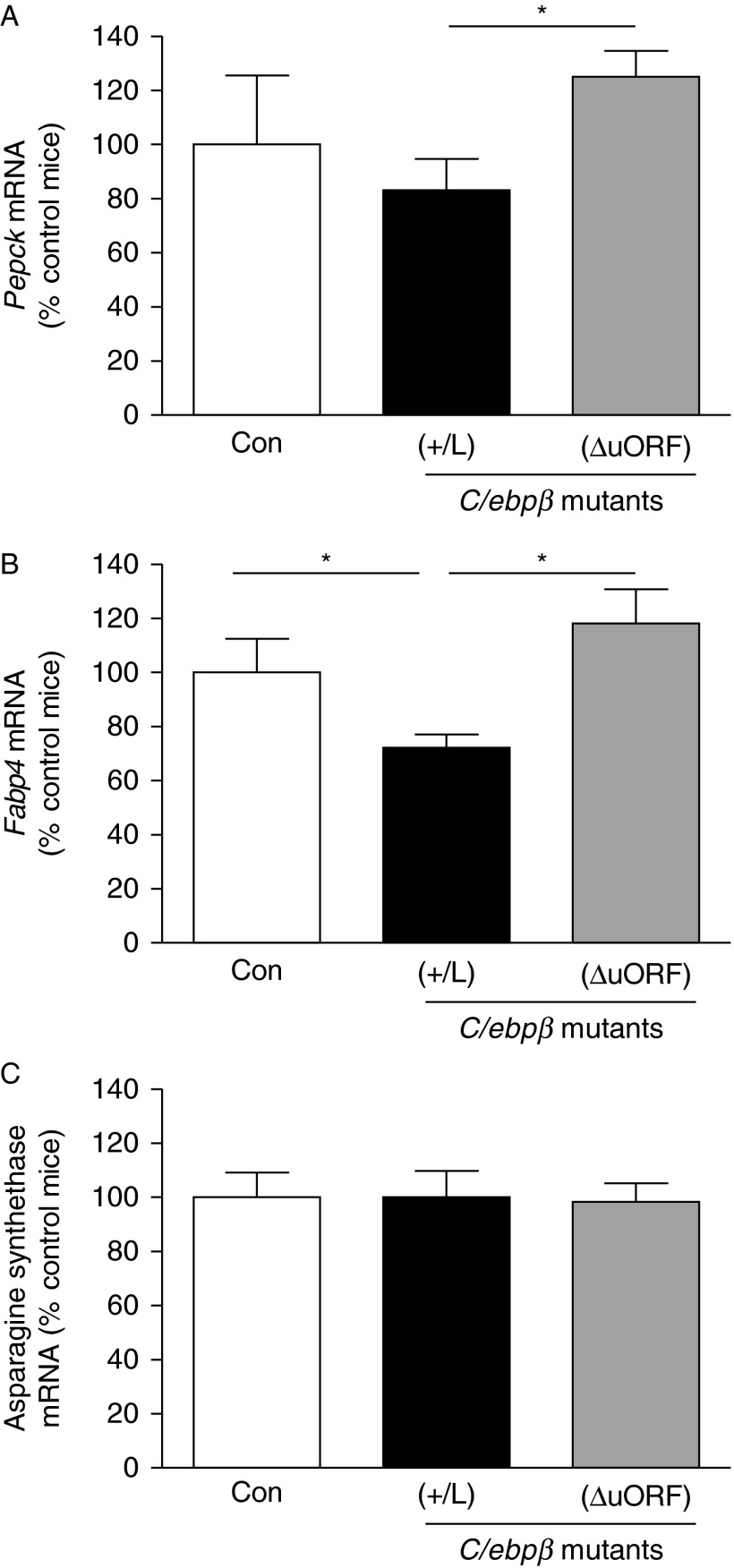
Elevated C/EBPβ-LIP represses expression of PEPCK and FABP4, but not asparagine synthetase in adipose tissue *in vivo*. Real-time PCR measurement of levels of mRNA encoding (A) PEPCK, (B) FABP4 and (C) asparagine synthetase in adipose tissue of wild-type control mice (Con, white bars), C/EBPβ^(^^+^^/L)^ (+/L, black bars) and C/EBPβ^Δ^^uORF^ (ΔuORF, grey bars). C/EBPβ^(^^+^^/L)^ mice are heterozygous for an allele of C/EBPβ in which the normal gene has been replaced by C/EBPβ-LIP (a ‘knock-in’; Smink *et al*. 2009) and C/EBPβ^Δ^^uORF^ mice have a homozygous disruption of the upstream ORF (Wethmar *et al*. 2010). Adipose tissue mRNA levels, normalised to TBP, are expressed relative to levels in control mice (arbitrarily set to 100%) and are mean±s.e.m.; *n*=6–9/group. *P≤0.05.

To test the relevance of the 3T3-L1 pSLIK-C/EBPβ-LIP adipocyte model to the *in vivo* situation, we measured the levels of mRNA encoding PEPCK, FABP4 and asparagine synthetase in the adipose tissue of mice genetically engineered to produce altered levels of C/EBPβ-LIP, which we have previously shown to regulate adipose tissue 11β-HSD1 mRNA levels (Esteves *et al*. 2012). C/EBPβ^(^^+^^/L)^ mice, with a ‘knock-in’ of C/EBPβ-LIP (L allele) replacing the normal C/EBPβ gene, have increased C/EBPβ-LIP levels and therefore increased C/EBPβ-LIP:LAP ratio (Smink *et al*. 2009). In contrast, in C/EBPβ^Δ^^uORF^ mice, deletion of the upstream open reading frame (ΔuORF allele) prevents translation of C/EBPβ-LIP (Wethmar *et al*. 2010), resulting in decreased C/EBPβ-LIP:LAP ratio (Esteves *et al*. 2012). In agreement with the results obtained in the 3T3-L1 pSLIK-C/EBPβ-LIP adipocytes, levels of mRNA encoding PEPCK were lower in the adipose tissue of C/EBPβ^(^^+^^/L)^ mice compared with C/EBPβ^Δ^^uORF^ (Fig. 4A). However, there was no significant decrease in PEPCK mRNA levels in C/EBPβ^(^^+^^/L)^ mice compared with WT controls, suggesting a larger effect of C/EBPβ-LIP *in vitro* than *in vivo* or that compensation has occurred *in vivo* (e.g. through tissues other than adipose). FABP4 levels were lower in the adipose of C/EBPβ^(^^+^^/L)^ mice compared with control and C/EBPβ^Δ^^uORF^ mice, while asparagine synthetase was unchanged (Fig. 4B and C). Taken together, these results show that the 3T3-L1 pSLIK-C/EBPβ-LIP adipocytes mimic the *in vivo* regulation.

## Discussion

The use of the pSLIK system to conditionally express C/EBPβ-LIP in fully differentiated 3T3-L1 mature adipocytes allowed investigating its impact on the transcriptional levels of 11β-HSD1, PEPCK, FABP4 and asparagine synthetase in these cells. Although transfection of adipocytes with small RNAs (siRNA and shRNA) has recently been achieved using nanoparticles of virus-derived amphipathic peptides (Li *et al*. 2007, Esteves *et al*. 2012), transfection of vectors harbouring expression cassettes remains a challenge. Electroporation, used in some studies (Thurmond *et al*. 1998), causes high levels of adipocyte death, is inefficient, and requires large amounts of DNA (or RNA). Adenovirus transduction allows expression of exogenous genes in 3T3-L1 adipocytes (Gnudi *et al*. 1997, Boizard *et al*. 1998, Duong *et al*. 2002, Carlotti *et al*. 2004) but does not produce stable expression and can induce immune responses. Many genes expressed in adipocytes, including 11β-HSD1 (Chapman *et al*. 2009), are immunoresponsive (Shoelson *et al*. 2006). Lentiviral vectors can be used to stably transduce adipocytes but require production of high titres of virus (Carlotti *et al*. 2004, Liao *et al*. 2006). The pSLIK vector used in the current study was originally designed as an intermediate in lentiviral construction (Shin *et al*. 2006) and combines all components required for expression (Tet-ON) and selection (Neo, GFP) in a single plasmid vector. This allows for the transfection of the pSLIK-C/EBPβ-LIP vector into 3T3-L1 preadipocytes without the need for lentivirus and proves a highly efficient process. Here, we used DOX to restrict expression of C/EBPβ-LIP to fully differentiated 3T3-L1 adipocytes, but ectopic transgene expression can be initiated at any stage of 3T3-L1 adipocyte differentiation or even induced transiently. Moreover, it may prove useful for expression of shRNA in differentiated adipocytes, to conditionally knockdown gene expression. Contrary to standard lentiviral transduction of adipocytes, here, all adipocytes harbour the gene of interest and expression is achieved soon after induction with DOX.

Elevation of C/EBPβ-LIP in mature differentiated 3T3-L1 adipocytes concomitantly decreased PEPCK and 11β-HSD1 mRNA levels, suggesting direct regulation of 11β-HSD1 by C/EBPβ-LIP in adipocytes. Indeed, both genes are bound by C/EBPβ and this has been shown for 11β-HSD1 in several cell types (Arai *et al*. 2007, Sai *et al*. 2008, Yang *et al*. 2009), including adipocytes (Esteves *et al*. 2012), and for PEPCK in hepatoma cells (Park *et al*. 1993, Choudhury *et al*. 2011). PEPCK mRNA levels declined faster than other genes, possibly reflecting the short half-life of PEPCK (Tilghman *et al*. 1974) compared with 11β-HSD1 mRNA, for example (Balachandran *et al*. 2008). FABP4 mRNA levels were only affected after more prolonged elevation of C/EBPβ-LIP, suggesting that either the mRNA is very stable or regulation is indirect. However, C/EBPβ-LIP expression did not depress global transcription in adipocytes, as asparagine synthetase mRNA levels were unaffected by elevated adipocyte C/EBPβ-LIP both *in vitro* and *in vivo*. This contrasts with the HepG2 hepatoma cells where asparagine synthase is regulated by the C/EBPβ-LIP:LAP ratio (Siu *et al*. 2001) and might reflect differences in epigenetic regulation.

Finally, we note that this system may be of broader application to explore the role of other adipocyte relevant genes in fully differentiated adipocytes, providing insights into their effect on adipocyte physiology.
